# Nurses' perceptions of patient participation in the myocardial infarction pathway

**DOI:** 10.1002/nop2.544

**Published:** 2020-06-15

**Authors:** Elise Kvalsund Bårdsgjerde, Bodil J. Landstad, Torstein Hole, Magne Nylenna, Kari Hanne Gjeilo, Marit Kvangarsnes

**Affiliations:** ^1^ Department of Health Sciences in Ålesund Faculty of Medicine and Health Sciences NTNU – Norwegian University of Science and Technology Ålesund Norway; ^2^ Department of Health Sciences Mid Sweden University Sundsvall and Östersund Sweden; ^3^ Levanger Hospital Nord‐Trøndelag Hospital Trust Nord‐Trøndelag Norway; ^4^ Clinic of Medicine and Rehabilitation Møre og Romsdal Hospital Trust Ålesund Norway; ^5^ Department of Circulation and Medical Imaging Faculty of Medicine and Health Sciences NTNU – Norwegian University of Science and Technology Trondheim Norway; ^6^ Institute of Health and Society University of Oslo Oslo Norway; ^7^ Norwegian Institute of Public Health Oslo Norway; ^8^ Department of Cardiothoracic Surgery St. Olavs Hospital Trondheim University Hospital Trondheim Norway; ^9^ Department of Cardiology St. Olavs Hospital Trondheim University Hospital Trondheim Norway; ^10^ Department of Public Health and Nursing Faculty of Medicine and Health Sciences NTNU – Norwegian University of Science and Technology Trondheim Norway; ^11^ Møre og Romsdal Hospital Trust Ålesund Norway

**Keywords:** focus group, myocardial infarction, myocardial infarction care, nurse perception, nurse perspective, nurses, nursing, patient involvement, patient participation, qualitative research

## Abstract

**Aim:**

To explore nurses' perceptions of patient participation in different phases of the myocardial infarction pathway.

**Design:**

Qualitative design with a hermeneutical approach.

**Methods:**

Five focus groups were conducted at two hospitals, one with and one without percutaneous coronary intervention facilities, between February–November 2018. Participants were recruited through purposive sampling. Twenty‐two nurses experienced in cardiac care participated. The analysis had a hermeneutical approach.

**Results:**

The findings revealed nurses' perceptions of patient participation in different phases of the myocardial infarction pathway. Four themes were identified: (a) variation between paternalism and autonomy in the acute phase; (b) individualization of dialogue and patient participation during treatment; (c) lack of coherence in the pathway hinders patient participation at discharge; and (d) cardiac rehabilitation promotes patients' autonomous decisions in lifestyle changes.

## 1 INTRODUCTION

Patient participation is a core element in patient‐centred care (Kitson, Marshall, Bassett, & Zeitz, 2013) and can improve patient safety and quality in health care (Vahdat, Hamzehgardeshi, Hessam, & Hamzehgardeshi, 2014; WHO, 2013). In most developed countries, patient participation is considered a legal right and a healthcare standard. Nurses have a key role in promoting patient participation (Angel & Frederiksen, 2015; Tobiano, Bucknall, Marshall, Guinane, & Chaboyer, 2015).

Treatment of myocardial infarction (MI) follows a standardized pathway which is divided into four phases: acute phase; treatment phase; discharge phase; and rehabilitation phase. An MI requires urgent treatment with antithrombotic medications and percutaneous coronary intervention (PCI). The urgency of PCI is dependent on the type of MI. For non‐ST‐elevation myocardial infarction (nSTEMI), the European Society of Cardiology (ESC) guidelines recommend PCI within 2–72 hr, dependent on the ischaemic risk, while for ST‐elevation myocardial infarction (STEMI), the recommendation is within 120 min (Ibanez et al., 2017; Roffi et al., 2015). PCI facilities are generally centralized to high‐volume centres for invasive treatment (Neumann et al., 2018). Norway has eight hospitals with PCI facilities. Therefore, patients often are transferred between hospitals to receive treatment (Hagen, Häkkinen, Belicza, Fatore, & Goude, 2015). Patients with MI being transferred between different hospitals have experienced the pathway as unplanned where the various hospitals were perceived as uncoordinated (Valaker et al., 2017).

Even if an MI is characterized as an acute event, it is caused by coronary artery disease (CAD), which is a chronic condition related to several risk factors, such as high blood pressure, high cholesterol levels, overweight and tobacco use. Therefore, secondary prevention with medication and lifestyle changes to reduce risk factors is necessary to prevent new cardiac events (Piepoli et al., 2016). Short hospital stays allow limited time for the initiation of secondary prevention (Ibanez et al., 2017; Roffi et al., 2015).

Patients are recommended to attend cardiac rehabilitation after discharge (Ibanez et al., 2017). Yet, participation rates in cardiac rehabilitation are low (Kotseva et al., 2016; Olsen, Schirmer, Bønaa, & Hanssen, 2018). Jortveit et al. (2019) found that risk factor control after MI was low; on average, three of six defined treatment targets for secondary prevention were achieved. Patient participation can increase patient motivation and responsibility for adhering to secondary prevention (Kähkönen et al., 2015; Thompson, 2007).

### 1.1 Background

Patient involvement and participation are often used synonymously. Thompson (2007) distinguished the two terms, defining involvement as a precondition for participation. Participation means that patients are engaged in discussions, provided with relevant information, asked about their opinions and participating in decision‐making processes (Thompson, 2007). Patient participation is attached to the ethical principle of autonomy. Autonomy is dependent on both the patient's competence and the context (Beauchamp & Childress, 2013).

Thompson, Ruusuvuori, Britten, and Collins (2007) provided an approach to understand patient participation based on three elements: components, levels and contexts. The components are divided into five different areas where the patient can participate: (a) contribution to action by initiation or responding in consultations; (b) defining the problem; (c) participation in the reasoning process; (d) participation in decision‐making; and (e) emotional reciprocity in encounters with healthcare professionals. These components are related to levels of involvement defined in Thompson's (2007) taxonomy. The levels follow a continuum from no involvement, to information‐seeking/reception, to information‐giving and dialogue, to shared decision‐making, to autonomous decision‐making (Thompson, 2007). In this study, we have used Thompson et al.'s (2007) integrative and dynamic approach to patient participation as a theoretical framework.

Previous research has found that patients with MI did not wish to participate in treatment decisions during the acute phase (Arnetz & Arnetz, 2009; Decker et al., 2007; Höglund, Winblad, Arnetz, & Arnetz, 2010; Radcliffe, Harding, Rothman, & Feder, 2009; Sampson, O'Cathain, & Goodacre, 2009). Later, when situations were stabilized and until discharge, patients' desires to participate increased (Arnetz & Arnetz, 2009; Decker et al., 2007).

Patients have reported a lack of information about secondary prevention (Astin, Closs, McLenachan, Hunter, & Priestley, 2008; Oterhals, Hanestad, Eide, & Hanssen, 2006; Valaker et al., 2017). Pettersen et al. (2018) found that patients perceived information about medications as insufficient.

The first period after discharge has been reported by patients as difficult (Astin et al., 2008; Junehag, Asplund, & Svedlund, 2014). Patients have reported that participating in cardiac rehabilitation enhanced their knowledge about their medical condition and increased their motivation to secondary prevention (Bårdsgjerde, Kvangarsnes, Landstad, Nylenna, & Hole, 2019; Valaker et al., 2017).

Previous research from a healthcare professional perspective has found that nurses considered patient information and participation as important in the MI pathway (Arnetz, Winblad, Arnetz, & Höglund, 2008; Arnetz & Zhdanova, 2015; Höglund et al., 2010). Arnetz and Zhdanova (2015) found that although patient participation was considered important, it did not necessarily result in a behaviour that facilitated participation. Furthermore, Arnetz et al. (2008) found that only 44% of the nurses and 62% of the physicians in their sample discussed lifestyle changes with patients before discharge.

An acute setting, short and fragmented hospital stay can hinder patient participation (Eldh, Ehnfors, & Ekman, 2004; Thompson, 2007; Valaker et al., 2017), and it has been identified that patients and healthcare professionals often have different perceptions of patient participation (Eldh, 2019; Höglund et al., 2010). Patients have reported lack of information and participation in different phases of the MI pathway (Bårdsgjerde et al., 2019). By exploring nurses' perceptions of patient participation in the MI pathway, we can gain new knowledge that can improve patient participation in clinical care. Therefore, the aim of the study was to explore nurses' perceptions of patient participation in the MI pathway. The research question was: What are nurses' perceptions of patient participation in different phases of the MI pathway?

## 2 THE STUDY

### 2.1 Design

This study had a qualitative design with a hermeneutical approach (Alvesson & Sköldberg, 2018; Gadamer, 2004; Howell, 2013). A hermeneutical approach is useful when the purpose is to seek, understand and interpret the underlying meaning of a concept in reference to a specific context (Alvesson & Sköldberg, 2018; Patton, 2015).

A hermeneutical inquiry involves interpretation and understanding based on two basic principles: an alternation between the parts and the whole, where the parts can be understood only from the whole and the whole can be understood only from the parts; and an alternation between pre‐understanding and understanding. A pre‐understanding is necessary to be open to and provide questions, and the resulting answers provide new insight for a new understanding (Alvesson & Sköldberg, 2018; Gadamer, 2004).

### 2.2 Participants

Purposive sampling was used to recruit participants to the study (Tong, Sainsbury, & Craig, 2007). Both female and male nurses at different ages, with differences in education and length of work experiences, were included to increase variation and diversity (Polit & Beck, 2017). The inclusion criteria were that the nurses: (a) worked in cardiac care and (b) had at least 1 year of experience in cardiac care.

To recruit nurses, we contacted two hospitals in mid‐Norway. Nurses at the two hospitals were invited because they were responsible for patient care in different phases of the MI pathway. Mostly, the nurses at the hospital without PCI facilities were responsible for patient care in the acute phase and rehabilitation phase. The nurses at the hospital with PCI facilities were responsible for patient care during the PCI treatment and at discharge. Nurses who met the inclusion criteria were invited face to face to participate in the study (Tong et al., 2007). Twenty‐two nurses participated, including three men and 19 women aged from 24–58 years. The demographic data are presented in Table 1.

**Table 1:** Demographic data

Demographic data	Participants (*N* = 22)
Age (years)	
21–30	5
31–40	7
42–50	8
52–60	2
Education	
Bachelor of nursing^a^	22
Specialized in cardiac nursing^b^	9
Specialized in intensive care nursing^c^	2
Master's degree in advanced clinical nursing^d^	1
Working place	
Emergency unit	3
Cardiac ward	13
Catheterization angiography laboratory	6
Outpatient cardiac rehabilitation clinic^e^	1
Experience as a nurse (years)	
1–5	4
6–10	5
11–15	3
16–21	7
>21	3
Experience with cardiac patients (years)	
1–5	4
6–10	5
11–15	3
16–21	9
>21	1

^a^ Bachelor's degree in Nursing (180 ECTS credits).

^b^ Further postgraduate education in cardiac nursing (60 ECTS credits).

^c^ Further postgraduate education in intensive care nursing (90 ECTS credits).

^d^ Master's degree in advanced clinical nursing (120 ECTS credits).

^e^ Shared position in a cardiac ward and outpatient cardiac rehabilitation clinic.

### 2.3 Data collection

Focus groups were conducted to understand the insights and experiences of individuals through conversations and exchanges of experiences (Krueger & Casey, 2015). A questioning route (Krueger & Casey, 2015) based on the aim of the study, previous research and the theoretical framework was developed. The questioning route consisted of open‐ended questions (Table 2). The question route was not used in a rigid way, and follow‐up questions were asked when needed.

**Table 2:** Question route

What experiences do you have with providing information in the myocardial infarction pathway?
How have you experienced patient participation in the myocardial infarction pathway?
Can you summarize the challenges with information provision and patient participation in the myocardial infarction pathway?
How can patient information and participation be strengthened in the myocardial infarction pathway?

Focus groups 1, 2 and 3 were conducted at the hospital with PCI facilities, and each group consisted of nurses working in the catheterization laboratory and various cardiac wards. Focus groups 4 and 5 were conducted at the hospital without PCI facilities. Focus group 4 consisted of nurses working at an emergency unit, cardiac ward and in cardiac rehabilitation, while focus group 5 consisted of nurses working at a cardiac ward. The size of the focus groups varied from three–five participants.

The focus groups were conducted in meeting rooms at the hospitals between February–November 2018. During the interviews, the participants were engaged in the topic and openly shared and exchanged experiences and opinions with each other. Each interview lasted approximately 90 min. The interviews were led by a moderator, while a co‐moderator observed the interactions in the groups and took notes. The interviews were audio‐recorded and transcribed verbatim. After five focus groups, we identified patterns and preliminary themes across the interviews and therefore considered the data to be saturated (Krueger & Casey, 2015).

### 2.4 Ethical considerations

The Norwegian Centre for Research Data approved the study. Prior to the interviews, the participants were informed in both oral and written formats and provided their written consent. The participants were informed that they could withdraw from the study without providing any reason. The participants were asked to anonymize examples and histories used during the interviews and to keep the content of the focus group confidential.

### 2.5 Data analysis

The analysis was guided by the study aim, research question (Krueger & Casey, 2015) and the theoretical framework (Thompson, 2007; Thompson et al., 2007). Our analysis was inspired by the two hermeneutical principles to generate new insights and understanding based on interpretations (Gadamer, 2004).

The analysis was performed by the first and the last authors. First, each interview was read in its entirety to gain insight into the content. Then, data were collated into initial codes related to the different phases of the pathway: acute phase, treatment phase, discharge phase and rehabilitation phase. We used our pre‐understanding, based on the theoretical framework, to question the data to identify discussions, meanings and expressions of patient participation (Gadamer, 2004). When the data were coded with the initial codes, we alternated between the parts of the pathway and the whole pathway in each interview and across the interviews. To collate the data into preliminary themes, we looked for patterns and diversity across the interviews. To structure the preliminary themes, we created subthemes for each theme. Then, each theme was abstracted and written in full, illustrated by suitable quotes, as shown in Table 3.

**Table 3:** Development of quotes into themes

Quotes	Subthemes	Theme
THEME 1		
“Sometimes it is critical; you do not delay an angiography because the patient should be very well informed” (N4, fg 3)	Severity of illness and lack of time prevent participation	
“It seems to be a reassurance for the patients that the physician confirms the information that we have already given” (N1, fg 4)	Providing consistent and concrete information	Variation between paternalism and autonomy in the acute phase
“It is important that elderly people have the possibility to say, ‘I do not want any invasive treatment. Let me live in peace the last years of my life’” (N4, fg 2)	Elderly patients often take autonomous decisions	
THEME 2		
“Some will know everything; others do not want to know anything. Some will look at the screen; others will not. And some of them will just have it done without any questions” (N4, fg 1)	Individual information tailored to patients' preferences	
“I experience that the patients often are engaged and ask what they can do themselves to prevent another event” (N2, fg 2)	Acute illness increases patients' receptivity for secondary prevention	Individualization of dialogue and patient participation during treatment
“Sometimes it [the MI] cannot be treated with PCI, and then we have to give that information and tell them that it will be discussed in the heart team and that they will receive more information later about treatment options: surgery or PCI” (N2, fg 3)	Patients' lack of medical knowledge hinders shared decision‐making in treatment	
THEME 3		
“First, they are admitted, then they go to the catheterization laboratory and then to the intensive care unit until the evening before they are coming back to the cardiac ward. And often, the next day, they are discharged” (N3, fg 2)	Fragmented and short pathway	
Dialogue from focus group 4: N3: “I do not think it is that stupid to have checklists for information that should be given before discharge. If you have checklists, it is easier to have things done.” N2: “At least for those with less experience that might be unsure about what information they are supposed to give.” N1: “The trouble is that there are so many schemes and checklists.”	Lack of routines and interprofessional collaboration in discharge planning	Lack of coherence in the pathway hinders patient participation at discharge
“If the patients are supposed to take part in decisions, it requires a great deal of information and that the patients really understand the information they have received” (N1, fg 3)	Shared decision‐making requires patient competence	
THEME 4		
“We revised the pamphlet and added information about the first period at home after an MI” (N5, fg 1)	Patients lacking information after discharge	
“I often say, ‘If you believe that you are going to live a normal life again, it is smart to take your wife with you to the cardiac rehabilitation so she can hear that you are going to live like normal’” (N4, fg 3)	Involving the spouse in cardiac rehabilitation	Cardiac rehabilitation promotes patients' autonomous decisions in lifestyle changes
“We cannot make changes if the patients do not take part in it” (N1, fg 4)	Patient engagement and involvement in cardiac rehabilitation	

### 2.6 Rigour

To enhance credibility, the method has been transparently described and the quotations were chosen carefully to substantiate the results (Lincoln & Guba, 1985). The co‐moderator provided a summary of the main points at the end of each interview, and the participants were invited to provide comments or corrections (Krueger & Casey, 2015). To ensure confirmability (Lincoln & Guba, 1985), three of the other authors also read the transcripts and the findings were discussed. Three of the authors had specific clinical experience in cardiac care. The findings are presented through rich descriptions to increase transferability (Lincoln & Guba, 1985).

## 3 RESULTS

Four themes related to the 22 nurses' perceptions of patient participation in different phases of the pathway were identified: (a) variation between paternalism and autonomy in the acute phase; (b) individualization of dialogue and patient participation during treatment; (c) lack of coherence in the pathway hinders patient participation at discharge; and (d) cardiac rehabilitation promotes patients' autonomous decisions in lifestyle changes.

### 3.1 Variation between paternalism and autonomy in the acute phase

In the acute phase, the nurses reported that the severity of the situation made patient information challenging. The nurses described that they worked in teams with physicians and that it was of great importance to monitor patients and initiate the correct treatment: “Sometimes it is critical; you do not delay an angiography because the patient should be very well informed” (N 4, fg 3).

The nurses noted that most of their patients were admitted to the cardiac ward to be prepared for angiography. The nurses gave information in both oral and written formats to prepare patients before angiography and PCI. This was perceived as challenging for the patients. The patients were often not able to participate in their care and treatment, as stated by one nurse, “They are not receptive to much information; their focus is often here and now” (N2, fg 5). The nurses reported that they often needed to repeat information several times. The nurses at the hospital without PCI facilities told that the patients often were more concerned about the transfer to the PCI hospital than the PCI procedure itself.

The nurses perceived it as difficult for the patients to grasp essential information and that they often did not understand the severity of their conditions. The nurses explained that collaborating with physicians about information in this phase was important. Consistent information from both physicians and nurses was considered important, as stated by one nurse: “It seems to be a reassurance for the patients that the physician confirms the information that we have already given” (N 1, fg 4).

The nurses described that in encounters with older and fragile patients, they often observed that these patients expressed a desire to participate in decisions about treatment, as illustrated by the following example: “It is important that elderly people have the possibility to say, ‘I do not want any invasive treatment. Let me live in peace the last years of my life’” (N 4, fg 2). The nurses discussed how the patients' preferences were accounted for in planning the treatment. The nurses highlighted a special need for attentiveness to make sure that the patients really understood the consequences of their decisions.

### 3.2 Individualization of dialogue and patient participation during treatment

Nurses working at the catheterization laboratory expressed that patients' conditions ranged from being fully awake and well informed to critically ill with reduced consciousness. When taking care of the most critical patients, the nurses reported that their focus was to keep the situation calm: “It is most important to give concise and clear information about what we are going to do and make sure that they are in safe hands” (N 1, fg 2). The nurses explained that patients had different reactions during procedures: “Some will know everything; others do not want to know anything. Some will look at the screen; others will not. And some of them will just have it done without any questions” (N4, fg 1). They said that they tried to respect and adjust the level of information provided during the procedure.

The nurses experienced some patients as being receptive to information in the treatment phase and that such patients often had questions about secondary prevention; as one nurse explained, “I experience that the patients often are engaged and ask what they can do themselves to prevent another event” (N2, fg 2). The nurses stated that the time and setting of a procedure might not be the best circumstances for dialogue. Nevertheless, the nurses expressed that if patients were motivated, they talked about secondary prevention alongside the procedure. As one nurse said, “We have the potential to guide people in the right direction” (N1, fg 2).

The nurses observed that physicians during the PCI procedure asked patients to give their consent before they implanted any stents. Nevertheless, the nurses revealed that patient participation was difficult to achieve in such situations, as the time was limited, and patients lacked knowledge to take part in decisions about treatment.

The nurses recounted that relaying information to patients was challenging when an angiography showed severe multivessel disease. Patients were not receptive to in‐depth information at this stage; instead, the nurses described giving patients a small amount of information: “Sometimes it [the MI] cannot be treated with PCI and then we have to give that information and tell them that it will be discussed in the heart team and that they will receive more information later about treatment options: surgery or PCI” (N2, fg 3). The nurses expressed that it posed ethical challenges to balance information about the severity of the disease. They did not want to provide overwhelming information during PCI; therefore, they often experienced that the patients were not adequately informed about the severity.

### 3.3 Lack of coherence in the pathway hinders patient participation at discharge

The nurses experienced that the time from PCI until discharge was short and fragmented, which could lead to challenges in meeting with patients. One nurse described the limited time and fragmentation as follows: “First, they are admitted, then they go to the catheterization laboratory and then to the intensive care unit until the evening before they are coming back to the cardiac ward. And often, the next day, they are discharged” (N 3, fg 2). Collaboration with physicians and between different wards was considered important to ensure that the patient received consistent information. One nurse explained, “I think it is important that the patients experience a common thread in the information” (N1, fg 1).

The nurses told that how involved the patients were in secondary prevention, such as control of risk factors, lifestyle changes and medication, varied. The nurses did not have standardized routines for what information the patient should receive before discharge. The nurses discussed whether they would have benefitted from checklists for providing patients with information. While some thought the use of checklists could be beneficial for patient safety and quality in the healthcare system, others thought it would be just another instance of increased bureaucracy. This is illustrated by a dialogue (fg 4):

N3: “I do not think it is that stupid to have checklists for information that should be given before discharge. If you have checklists, it is easier to have things done.”

N2: “At least for those with less experience that might be unsure about what information they are supposed to give.”

N1: “The trouble is that there are so many schemes and checklists.”

The nurses observed that patients' levels of receptiveness to secondary prevention varied. They noted that participation in medical decisions required that the patient had enough competence; as one nurse stated, “If the patients are supposed to take part in decisions, it requires a great deal of information and that the patients really understand the information they have received” (N1, fg 3). The nurses explained that patients' lack of medical knowledge could be a barrier to shared decision‐making and that patients' lack of medical knowledge often led to healthcare professionals making decisions on behalf of patients. As one nurse explained, “We do not ask the patients whether they are interested or not in taking their prescribed medications” (N 4, fg 3).

Although participation in decisions was reported to be difficult, one situation was described differently, namely, when an angiogram showed multivessel disease that could be treated by PCI or bypass surgery. One nurse described the situation as follows: “When we consider which option is the best, bypass or several stent, we always listen to the patient's opinions and motivations” (N4, fg 3). In the focus group discussions, shared decision‐making was especially emphasized in these situations, when the severity of disease made the decision challenging.

The nurses explained that it was usually physicians who gave information to the patients at discharge. The nurses expressed that they would have preferred to be more involved in this process, but that time and resource constraints made their greater involvement impossible. Nurses revealed that they spent a substantial amount of time organizing the journey home, as described by one nurse, “It is not easy to get people back home in the rural areas that you are not familiar with yourself” (N 5, fg 1).

### 3.4 Cardiac rehabilitation promotes patients' autonomous decisions in lifestyle changes

The nurses were concerned for their patients after discharge because they knew that patients found the first period at home to be difficult and often needed information. Therefore, they had developed a pamphlet with information about each phase of the pathway, which had been recently revised: “We revised the pamphlet and added information about the first period at home after an MI” (N5, fg 1). Nevertheless, they reported that a well‐known problem was that the patients left the pamphlets behind at discharge.

The nurses revealed that they felt the patients did not receive enough information about their medications. They explained that even if they focused on the importance of adherence to medications during the hospital stay, they still observed that patients were readmitted because they had ceased taking their medications.

Before discharge, the nurses asked the patients if they wanted to attend in cardiac rehabilitation at their local hospital. They tried to encourage the patients to attend. Nevertheless, the nurses observed that the patients who they considered to need the programme most often declined the offer.

The nurses explained that next of kin were also invited to the cardiac rehabilitation, which they considered important because next of kin did not always receive information at the hospital. One nurse noted: “I often say, ‘If you believe that you are going to live a normal life again, it is smart to take your wife with you to the cardiac rehabilitation so she can hear that you are going to live like normal’” (N 4, fg 3). The nurses expressed that involving the spouse could have a positive impact on adherence to treatment.

One experienced nurse described patient participation as essential in cardiac rehabilitation. The nurse explained how they worked individually with each patient, going carefully through their risk factors and medications, and making sure that the patients truly understood everything. Nevertheless, the nurse emphasized that they were dependent on the patient's engagement: “We cannot make changes if the patients do not take part in it” (N1, fg 4). The nurse described patient engagement as crucial to achieving treatment adherence.

## 4 DISCUSSION

The aim of this qualitative study was to explore nurses' perceptions of patient participation in different phases of the MI pathway. We determined that the level of patient participation differed both between phases and within phases due to the specific contexts.

Consistent with ESC guideline recommendations (Neumann et al., 2018), the nurses in our study revealed that priority was given to initiate treatment in the acute phase. Beauchamp and Childress (2013) argue that healthcare professionals often behave paternalistically out of beneficence and clear guidelines often support healthcare professionals to act with the intentions of doing what is best for the patients. The nurses reported that they provided patients with information but that patients often were not receptive to information in the acute phase. Hospital transfers were perceived by the nurses as an obstacle for patient information. Consistent and clear information from both nurses and physicians was considered important in this phase, which is in accordance with patients' preferences (Decker et al., 2007; Höglund et al., 2010).

However, notably, the nurses in our study revealed that older patients often declined invasive treatment and made autonomous decisions about their own treatment. Previous studies have found that in general, older patients compared with younger patients more seldom participate (Angel & Frederiksen, 2015; Arnetz & Arnetz, 2009; Vahdat et al., 2014), and therefore, this finding provides new insight into a context that might be different from what has been assumed. Nevertheless, similar findings were documented in a study exploring patients' preferences for treatment, where older patients suffering from angina often preferred treatment with medication over invasive treatment options (Bowling, Culliford, Smith, Rowe, & Reeves, 2008).

Our study provides insight into how nurses working in a catheterization laboratory involved the patients through a dialogue based on the needs of each patient. A dialogue is described in Thompson's (2007) taxonomy as a precondition for patient participation. This dialogue seemed to be trigged of the patients' awareness of the severity of the situation making them motivated to prevent new cardiac events.

The nurses in our study told that the patients were asked to consent to the treatment during the procedure, yet they did not label the consent as a form of participating in decisions. This is supported by Beauchamp and Childress (2013), who claim that informed consent should not be equalized with shared decision‐making. An especially challenging ethical context was when multivessel disease was detected during angiography and treatment decisions needed to be discussed. The nurses emphasized that to discuss treatment options with the patients was important and that the final decision should be made based on the patients' preferences. This finding is consistent with previous studies that have found that patients often have preferences for treatment with medications, PCI or bypass surgery (Bowling et al., 2008; Doll et al., 2019).

Several barriers to participation at discharge were identified in our study. The pathway was described as short and fragmented. A mutual relationship where the patient and healthcare professionals experience emotional reciprocity is a prerequisite for patient participation (Thompson, 2007; Thompson et al., 2007), and fragmentation seemed to be a barrier in building such relations between the patient and healthcare professionals. Furthermore, a lack of routines made it difficult for the nurses to provide information, as they did not know what information patients had received earlier in the pathway. This finding is consistent with those of other studies that have stated that a lack of continuity and time are hindrances for patient participation (Angel & Frederiksen, 2015; Arnetz et al., 2008; Vahdat et al., 2014; Valaker et al., 2017).

Another finding was that when patients were not involved, whether due to organizational factors or patients' lack of knowledge, healthcare professionals often made decisions on behalf of the patients and then informed the patients afterwards. According to Thompson (2007), patient participation is dependent on the willingness of both patients and healthcare professionals. Healthcare professionals that out of beneficence exclude patients from taking part in their treatment may hinder patient participation. This finding gives us a deeper insight into the ethical challenges in the healthcare system that can explain why the level of participation is sometimes low or non‐existent.

Our findings showed that the nurses perceived that patient's lack of knowledge often was an obstacle to patient participation in treatment decisions. Health literacy means that the patient develops knowledge, skills and confidence to change their lifestyle and living condition (WHO, 2016). Previous research has found that patients do not reach their treatment targets after an MI (Jortveit et al., 2019). Good information may strengthen patients' health literacy. Health literacy is an important prerequisite for patient participation and adherence to secondary prevention.

In our study, the nurses were not greatly involved in planning discharge and preparing patients for their early rehabilitation. Instead, the nurses described being responsible for organizing the journey home for patients. Arnetz et al. (2008) revealed that nurses less often than physicians discussed lifestyle changes with patients before discharge and this can be an explanation of how tasks are divided between nurses and physicians. There is a need to discuss how nurses' resources are distributed and whether the responsibility for planning the journey home should be placed in the nurse profession. Further, nurses and physicians should collaborate in preparing the patients for discharge, as both the nurses in our study and previous research have stated that patients lack information at discharge (Arnetz et al., 2008; Astin et al., 2008; Decker et al., 2007; Oterhals et al., 2006; Pettersen et al., 2018; Valaker et al., 2017).

As the MI pathway was described as short and fragmented, the nurses in our study highlighted the need for cardiac rehabilitation. In line with recommendations (Ibanez et al., 2017), the nurses invited patients to participate in cardiac rehabilitation. Previous research has found that participating in cardiac rehabilitation is crucial for patients to enhance their health literacy and increase adherence to secondary prevention (Bårdsgjerde et al., 2019; Valaker et al., 2017). Although both healthcare professionals and patients agree that participating in cardiac rehabilitation is important, the participation rates are low (Kotseva et al., 2016; Olsen et al., 2018). Our findings reveal that the nurses experience that it is the most motivated patients that want to attend in cardiac rehabilitation. A possible solution to increase participation rates could be to automatically refer all patients to cardiac rehabilitation. However, such a solution would not promote patient participation in decision‐making, and as emphasized in our study, the key to successful cardiac rehabilitation is patients' own engagement. Findings from our study revealed that patient participation was best provided in cardiac rehabilitation, which the nurses described as providing continuity and individualization of care and treatment.

### 4.1 Limitations

The interviews were conducted and transcribed in Norwegian. First translation of quotes from Norwegian to English was done by the authors. A text editing service was used to scrutinize the text.

A hermeneutical interpretation can never be absolute and must remain an interpretation (Patton, 2015). The understanding of the interviews took place in a process where the meaning of the separate parts was determined by the global meaning of the interviews (Alvesson & Sköldberg, 2018; Gadamer, 2004). The interpretation of the interviews was based on communicative validation among the researchers (Kvale & Brinkmann, 2009).

Using a theoretical framework (Thompson, 2007; Thompson et al., 2007) may be a limitation. However, the chosen theoretical framework contributes to an understanding of the content and meaning of the term patient participation, which makes it explicit what has been studied.

## 5 CONCLUSION

This study provides new insight into nurses' perceptions of patient participation in the MI pathway. Patient participation varied in the different phases of the pathway. In the acute phase and during treatment, the nurses were committed to providing the right treatment. At discharge, the nurses revealed that the fragmented pathway and the lack of interprofessional cooperation hindered continuity in patient participation. We argue that there is a need to strengthen cooperation at the system level. In the rehabilitation phase, the nurses expressed that patient participation is essential to promote secondary prevention.

## CONFLICT OF INTEREST

No conflicts of interest have been declared by the authors.
